# Primary Sclerosing Cholangitis and Crohn's Disease: A Rare Association

**DOI:** 10.7759/cureus.69397

**Published:** 2024-09-14

**Authors:** Isha Bansal, Abhijeet Karad, Debabrata Banerjee, Amol S Dahale

**Affiliations:** 1 Medical Gastroenterology, Dr. D. Y. Patil Medical College, Hospital and Research Centre, Dr. D. Y. Patil Vidyapeeth (Deemed to be University), Pune, IND; 2 Gastroenterology and Hepatology, King Edward Memorial (KEM) Hospital and Seth Gordhandas Sunderdas (GS) Medical College, Mumbai, IND; 3 Gastroenterology and Hepatology, Command Hospital, Kolkata, IND

**Keywords:** crohn’s disease, granuloma, hematemesis, inflammatory bowel disease, primary sclerosing cholangitis

## Abstract

Inflammatory bowel disease (IBD) and primary sclerosing cholangitis (PSC) are closely associated disease entities that, when present in combination, create a phenotypically different summative disease referred to as PSC-IBD. The hallmark of Crohn's disease is persistent inflammation that can affect any part of the gastrointestinal system, from the mouth to the anus, although the terminal ileum and proximal colon are most frequently affected. Crohn's disease can sporadically impact the duodenum and stomach. Here, we present the case of a 12-year-old child who presented with per rectal bleed, hematemesis, and persistent elevated transaminasemia. A biopsy revealed micro-granulomas from the stomach's antrum and colonic mucosa, as well as focally exacerbated colitis. For a prolonged increased cholestatic pattern of liver enzymes, we performed an autoimmune panel and magnetic retrograde cholangiopancreatography, which revealed PSC. The patient was started on steroids and immunosuppressants.

## Introduction

Primary sclerosing cholangitis (PSC) was identified as one of the most pressing unmet needs in hepatology at the International Liver Congress [1]. Unfortunately, little has changed since then, owing to the disease's elusive pathogenesis. PSC is more commonly associated with inflammatory bowel disease (IBD), with most patients having a diagnosis of severe colitis. Whether PSC is only an extra-intestinal form of IBD or if PSC and IBD are two distinct conditions sharing a common tendency leading to a dual phenotype is a matter of debate. IBD and PSC are thought to have similar pathophysiology despite their exact pathogenesis being unknown. It is believed that immune-mediated mechanisms (including biliary epithelial cell abnormalities, immunogenic vulnerability, increased intestinal permeability, dysbiotic gut microbiota, and hereditary predisposition) have a role [1].

IBD, especially ulcerative colitis (UC), is frequently linked to underlying PSC. PSC is less likely to be related to Crohn's disease (CD) [1,2]. In contrast, PSC is diagnosed in 2%-14% of individuals with IBD, while IBD is present in 60%-80% of PSC patients. The PSC-CD phenotypic is quite different from the typical CD, just as PSC-UC. In particular, compared to rates of approximately 30% in conventional CD, solitary ileal involvement in PSC-CD is uncommon, with rates ranging from 2% to 5% [3]. As a result, PSC-CD had greater incidences of colitis and ileocolitis than CD alone. We describe the case of a young boy with bloody diarrhea and hematemesis who was later diagnosed with CD with the less common accompanying feature of PSC.

## Case presentation

A 12-year-old boy presented to the medical gastroenterology clinic with complaints of increased frequency of stools with blood and mucus, colicky abdominal pain, and vomiting with blood occasionally for four months. He also complained of mild fever and a history of significant weight loss and anorexia. Initially, he had one to two episodes of stools mixed with blood per day, which increased in frequency to three to four episodes per day. The pain in the abdomen was diffuse and colicky, aggravated by eating, and relieved on passing stool or after vomiting. There was no significant history of comorbidities or past surgery. There was also no similar history in the family. On initial examination, the patient was poorly built and nourished, with a pulse rate of 138 beats per minute, a respiratory rate of 24 cycles per minute, and a blood pressure of 94/66 mmHg. Temperature and oxygen saturation were normal. An abdomen examination revealed diffuse superficial tenderness, and bowel sound was heard in all quadrants. His fecal calprotectin came high at 425.67 μg/g (normal value: 50-200 μg/g). The patient was initially stabilized with intravenous fluids and ciprofloxacin antibiotics. His laboratory investigation showed low hemoglobin, a raised leukocyte count, raised liver enzymes, and stool showing significant red blood cells (Table 1).

**Table 1:** Laboratory investigations TLC: total leukocyte count; RBC: red blood cell

Investigation	Value	Normal Value
Hemoglobin	9.1 g/dL	11.6-15 g/dL
TLC	13,600 cells/μL	4000-10,000 cells/μL
Platelets	345,000 cells/μL	150,000-410,000 cells/μL
Total bilirubin	0.46 mg/dL	0.22-1.2 mg/dL
Conjugated bilirubin	0.22 mg/dL	0.1-1.0 mg/dL
Serum glutamic oxaloacetic transaminase	187 U/L	8-43 U/L
Serum glutamate pyruvate transaminase	177 U/L	7-45 U/L
Alkaline phosphatase	599 U/L	50-117 U/L
Gamma-glutamyl transferase	374	5-36 U/L
Urea	18 mg/dL	17-49 mg/dL
Creatinine	0.53 mg/dL	0.6-1.2 mg/dL
Serum albumin	2.7 g/dL	3.5-5.2 g/dL
Stool analysis	RBC many	RBC absent
Stool culture	No growth	No growth

**Figure 1:**
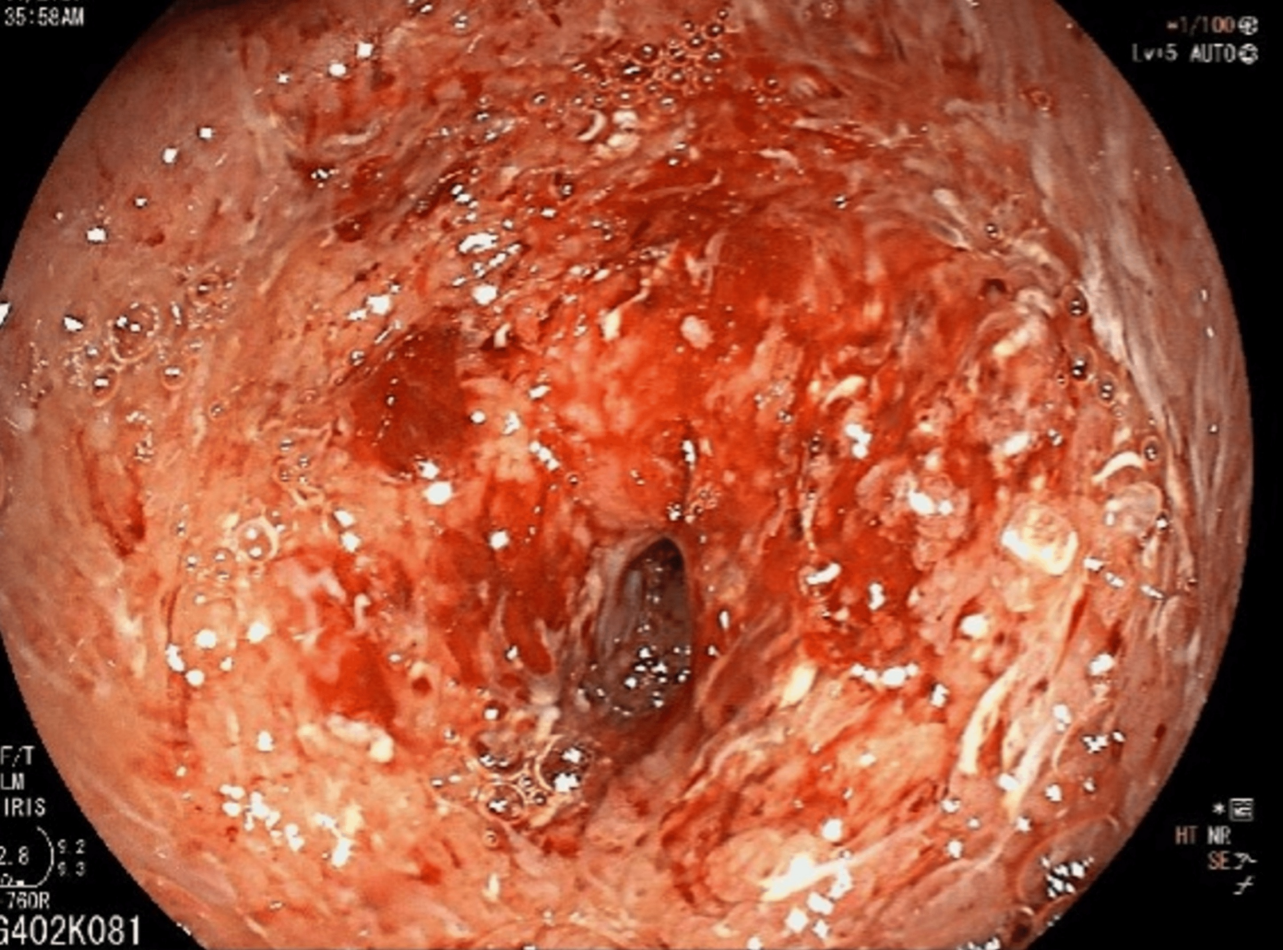
Esophago-gastroduodenoscopy showed edematous, erythematous mucosa with multiple superficial ulcerations in the fundus and antrum of the stomach.

An upper endoscopy showed edematous, erythematous mucosa with multiple superficial ulcerations in the fundus and antrum of the stomach. The antro-duodenal portion was deformed (Figure 1).

**Figure 2:**
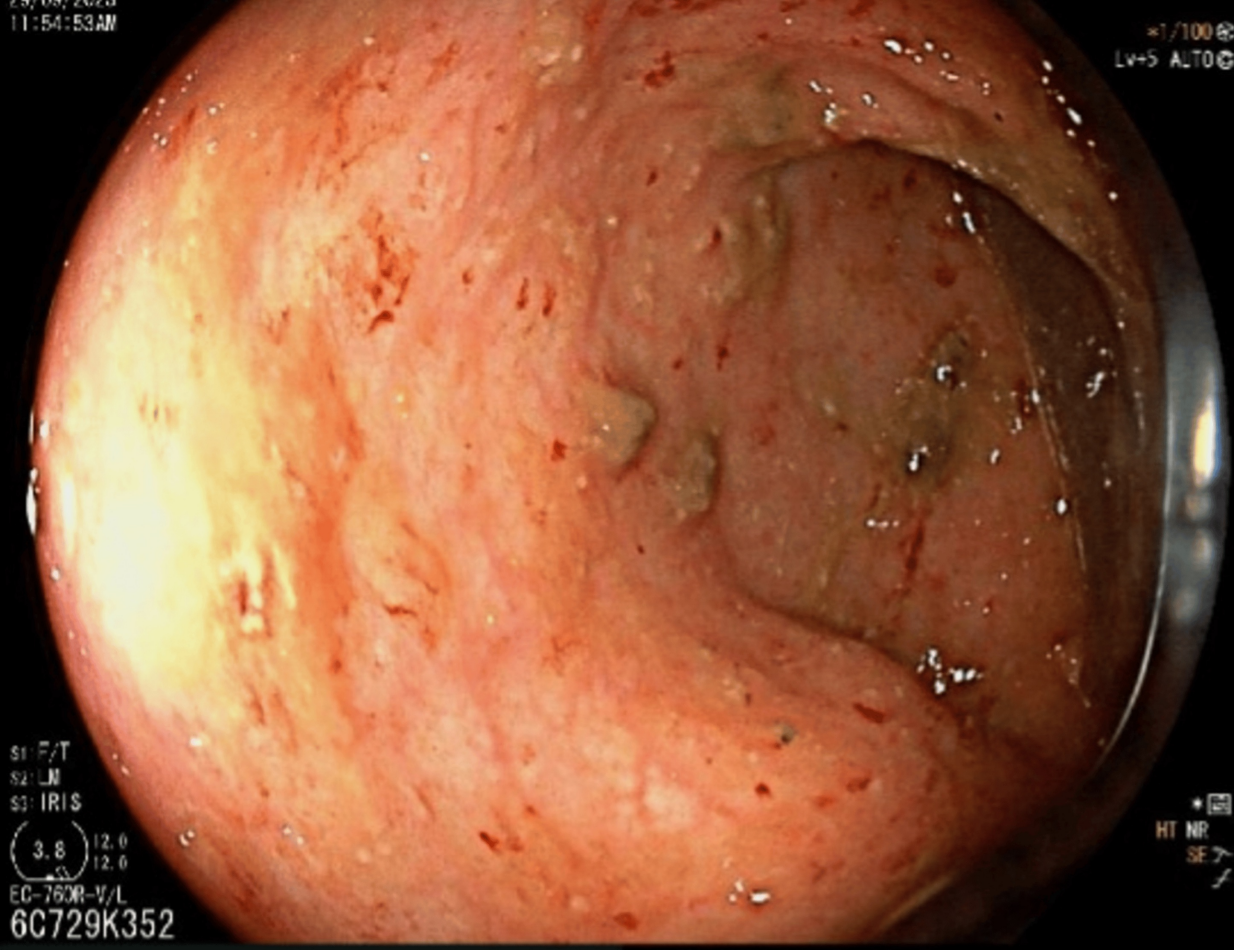
Colonoscopy image - the visualized mucosa shows edema, patchy erythema, and multiple superficial ulcerations.

A colonoscopy from the rectum to the transverse colon showed edema, patchy erythema, and multiple superficial ulcerations in the visualized mucosa. Erosions were noted at the ileocecal valve, while the terminal ileum mucosa appeared nodular and erythematous. The cecum and ascending colon showed a normal mucosa (Figure 2). Outside computed tomography enterography showed edematous wall thickening of the antrum, and enlarged enhancing non-necrotic mesenteric lymph nodes were observed in the right lower quadrant of the abdomen, with the largest measuring 10 mm in short-axis diameter.

**Figure 3:**
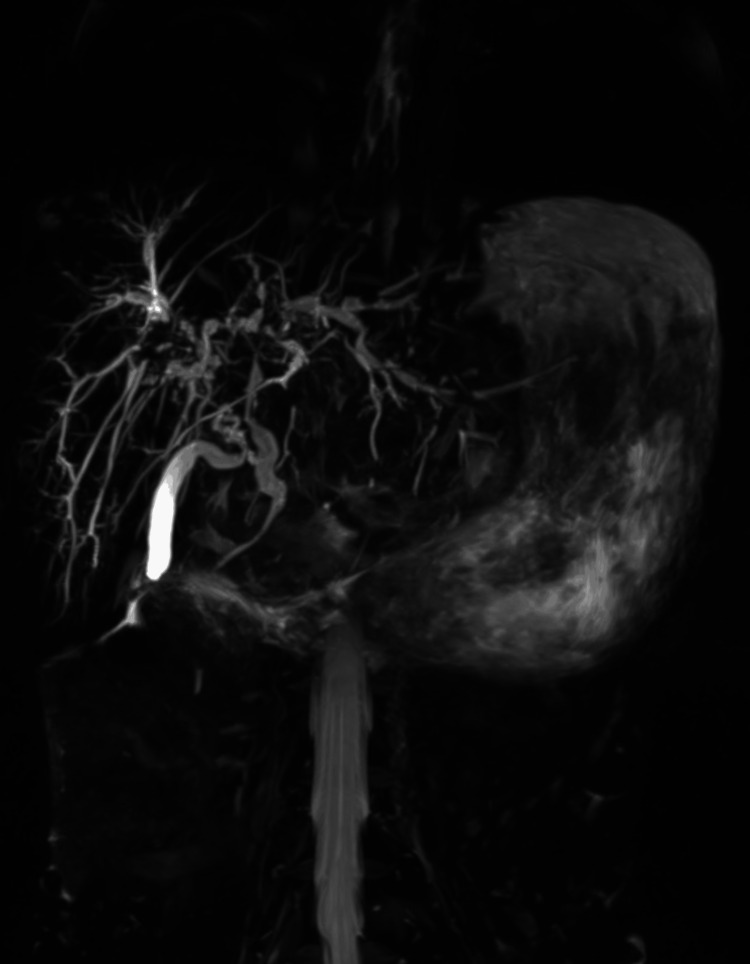
The MRCP revealed a circumferential wall thickness in the CBD and CHD, causing irregular segmental narrowing that extends proximally to the right and left hepatic ducts, along with mild dilatation of the IHBR in both lobes. MRCP: magnetic retrograde cholangiopancreatography; CBD: common bile duct; CHD: common hepatic duct; IHBR: intrahepatic biliary radical

Meanwhile, elevated transaminitis was worked up with further investigations. Hepatitis A virus (HAV)-IgM, Hepatitis E virus (HEV)-IgM, anti-nuclear antibodies-immunofluorescence (ANA-IF), anti-smooth muscle antibody (ASMA), anti-liver kidney microsomal antibody (anti-LKM), total immunoglobulin G, anti-mitochondrial antibody M2, and serum ceruloplasmin came out negative. Fundus examination revealed no Kayser-Fleischer ring. Elastography of the liver showed no fibrosis with a liver stiffness measurement of 4.7 kPa. A liver function test revealed persistent elevated alkaline phosphatase and gamma-glutamyl transferase (GGT) levels. The magnetic retrograde cholangiopancreatography (MRCP) on day five revealed a circumferential wall thickness in the common bile duct and common hepatic duct, causing irregular segmental narrowing that extends proximally to the right and left hepatic ducts, along with mild dilatation of the intrahepatic biliary radical in both lobes concerning for PSC (intra- and extrahepatic) (Figure 3).

Biopsies were taken during this colonoscopy and upper esophagoscopy for cytology. A colonic biopsy showed patchy crypt distortion, along with a patchy increase in lymphocytes and plasma cells. Focally enhanced colitis is observed, characterized by neutrophilic overrunning of crypts with surrounding histiocytes and eosinophils. The ileal mucosa showed normal villous crypt morphology. Hyperplastic lymphoid follicles are present. The lamina propria exhibits a mild increase in plasma cells along with a prominent increase in eosinophils. Occasional neutrophilic cryptitis is present with basal plasmacytosis.

**Figure 4:**
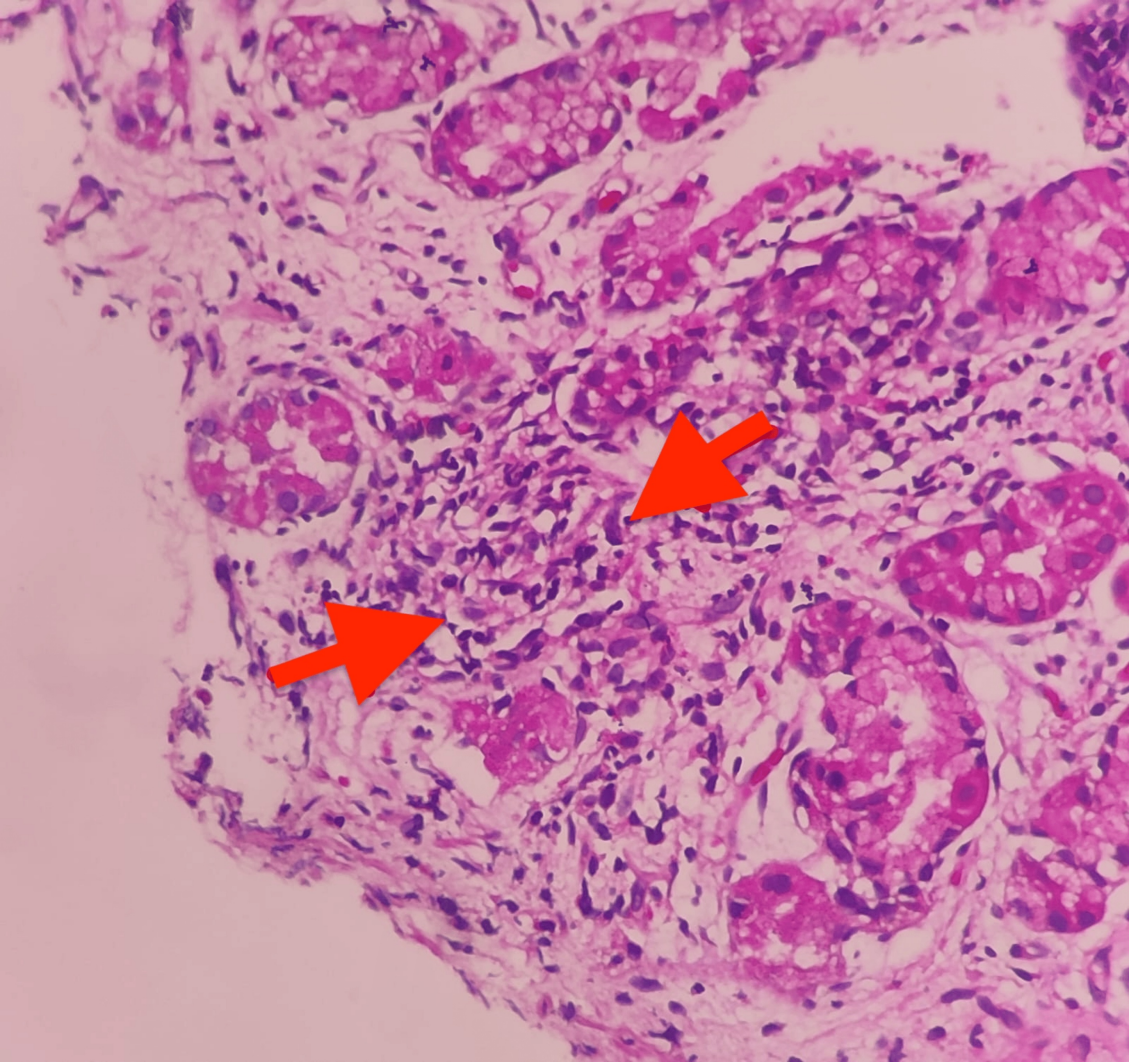
A gastric biopsy showed patchy neutrophilic pititis; occasionally, small microgranulomas (red arrows) composed of a few epithelioid cells were observed.

A gastric biopsy showed gastric antral and body type mucosa with foveolar hyperplasia and a patchy increase in chronic inflammatory cells and eosinophils. Patchy neutrophilic pititis was also seen. Occasionally, small microgranulomas composed of a few epithelioid cells were seen (Figure 4). Features were suggestive of IBD-CD.

The patient was treated with prednisolone 25 mg once a day and azathioprine 50 mg once a day. The prednisolone was tapered in a month, while azathioprine was continued. He showed improvement in his symptoms. He is currently undergoing regular follow-up and is asymptomatic.

## Discussion

A study conducted by Halliday et al. [4] indicated that PSC-CD less frequently proceeded to malignancy, liver transplantation, or death compared to PSC-UC. Only 32 CD patients were included in the study, leading the authors to hypothesize that the small sample size reduced the significance of the findings. To the best of our knowledge, there has been no direct comparison of the clinical outcomes of UC and CD patients in North America with related PSC in terms of the illness course, risk of colon neoplasia, or colectomy.

It has been suggested that the disease activity seen in each organ may affect the other because of the distinct phenotype of PSC-IBD. Colectomy was one intervention that seems to affect hepatic disease activity, according to recent studies on the connection between IBD disease activity and PSC outcomes. Two 2018 studies that focused on the effects of colectomy on PSC-IBD patients most likely shared patient population data. Early colectomy, even before PSC was diagnosed in more than 200 patients, was reported by Nordenvall et al. to be linked to a lower risk of death and eventual liver transplantation [5]. Additionally, Lindström et al. discovered that individuals with PSC-IBD who had colectomy before receiving a liver transplant exhibited a lower chance of developing PSC again. Nonetheless, it is important to note that this study's findings did not link the likelihood of recurrent PSC in the hepatic graft with the activity of the IBD illness [6].

## Conclusions

PSC is commonly associated with UC but can also occur in patients with CD. Patients presenting with both conditions require screening for colorectal neoplasia, cholangiocarcinoma, and gall bladder carcinoma.

It is currently believed that dysbiosis of the gut flora, abnormal microbiotic epitope recognition, and lymphocyte cross-reactivity influence the diseases. New biological medicines may enable the development of novel therapeutics for PSC-IBD by altering the interaction between the immune system and the target organs.
